# Review of Technological Challenges in Personalised Medicine and Early Diagnosis of Neurodegenerative Disorders

**DOI:** 10.3390/ijms24043321

**Published:** 2023-02-07

**Authors:** Celtia Domínguez-Fernández, June Egiguren-Ortiz, Jone Razquin, Margarita Gómez-Galán, Laura De las Heras-García, Elena Paredes-Rodríguez, Egoitz Astigarraga, Cristina Miguélez, Gabriel Barreda-Gómez

**Affiliations:** 1 Research and Development Division, IMG Pharma Biotech, 48160 Derio, Spain; 2 Department of Pharmacology, Faculty of Medicine and Nursing, University of the Basque Country UPV/EHU, 48940 Leioa, Spain; 3 Neurodegenerative Diseases Group, Biocruces Bizkaia Health Research Institute, 48940 Barakaldo, Spain

**Keywords:** biomarker, Parkinson’s disease, Alzheimer’s disease, imaging techniques, neuroinflammation, exosomes, beta-amyloid, reactive antibodies, alpha-synuclein

## Abstract

Neurodegenerative disorders are characterised by progressive neuron loss in specific brain areas. The most common are Alzheimer’s disease and Parkinson’s disease; in both cases, diagnosis is based on clinical tests with limited capability to discriminate between similar neurodegenerative disorders and detect the early stages of the disease. It is common that by the time a patient is diagnosed with the disease, the level of neurodegeneration is already severe. Thus, it is critical to find new diagnostic methods that allow earlier and more accurate disease detection. This study reviews the methods available for the clinical diagnosis of neurodegenerative diseases and potentially interesting new technologies. Neuroimaging techniques are the most widely used in clinical practice, and new techniques such as magnetic resonance imaging (MRI) and positron emission tomography (PET) have significantly improved the diagnosis quality. Identifying biomarkers in peripheral samples such as blood or cerebrospinal fluid is a major focus of the current research on neurodegenerative diseases. The discovery of good markers could allow preventive screening to identify early or asymptomatic stages of the neurodegenerative process. These methods, in combination with artificial intelligence, could contribute to the generation of predictive models that will help clinicians in the early diagnosis, stratification, and prognostic assessment of patients, leading to improvements in patient treatment and quality of life.

## 1. Introduction

Neurodegenerative disorders (NDs) are characterised by the gradual loss of certain groups of nervous system cells, accompanied by enhanced depositions of proteins with important functions in cellular homeostasis. These pathologies can be classified according to the protein accumulation [1], but they share common characteristics, such as the failure of molecular cleaning systems (ubiquitin–proteasomal and autophagosomal/lysosomal), excessive reactive oxygen species, neuroinflammation, and neuronal death [2].

Several well-known NDs involve intracellular or extracellular misfolded aggregates in different parts of the brain. These are formed by amyloid-beta (Aβ) in Alzheimer’s disease (AD), tau in AD and other tauopathies such as frontotemporal dementia, α-synuclein (α-Syn) in Parkinson’s disease (PD), Lewy bodies (LBs) in Lewy body dementia, and pri on proteins in prion diseases such as Creutzfeldt–Jakob disease, among others [3]. The functions of these proteins are very different. Aβ plays a key role in regulating signalling, neuronal homeostasis, development, and intracellular transport. Tau participates in signalling, synaptic plasticity, and microtubule stability in axons [4]. However, all these proteins can show misfolded or post-translational modifications that lead to aggregation, resulting in oligomeric or fibrillary structures [5]. The ways in which these proteins spread systemically in the brain have been thoroughly studied in both human and animal models, revealing recognisable patterns of spatial distribution in each pathology [6] (Table 1). The propagation of misfolding and the intercellular transfer of protein inclusions are similar to a prion protein transmission, prompting some NDs to be described as “prion-like” disorders.

The probability of protein aggregation and spreading, together with the gravity of the damage they cause to nervous system cells, depends on multiple risk factors that can be sporadic or genetic. One of the main risk factors in NDs is ageing. As molecular repair mechanisms and cleaning systems gradually become downregulated, it becomes more challenging to remove protein deposits [7]. The causes underlying these alterations remain unknown. There are cases in which NDs present genetic origins, but the vast majority of patients are idiopathic. Numerous risk factors for idiopathic NDs have been identified so far, and most of them relate to lifestyle issues such as obesity, failure in cholesterol homeostasis, stroke, traumatic brain injury, alcohol consumption, and poor diet. The brain is also sensitive to environmental factors such as pollution, the presence of heavy metals, vitamin deficiency, and electromagnetic radiation, which could be the cause of idiopathic cases [8]. Recently, exposure to microwave radiation has been related to cognitive impairment, affecting learning and memory processes [9]. Regarding genetics, a significant number of mutations in genes that play essential roles in homeostasis processes have been identified as causes (fully penetrant) or influences (incompletely penetrant) of NDs. Except for Huntington’s disease, NDs have complex aetiologies, displaying relatively rare genetic forms with early onset and, more frequently, multifactorial and idiopathic forms with late onset.

**Table 1:** Main features of the most prevalent neurodegenerative diseases.

Neurodegenerative Disease	Brain Origin	Physiological Characteristics	Most Vulnerable Neurons	Main Symptoms
**Alzheimer’s disease**	Brainstem nuclei, locus coeruleus, transentorhinal region, and olfactory bulb [10]	Hyperphosphorylated tau protein, β-amyloid plaques, neurofibrillary tangles, and neuritic plaques [11,12]	Cholinergic groups of neurons [13]	**Noncognitive symptoms:** aphasia, apraxia, agnosia, visuospatial deficits, difficulties in daily routine, and sleep disorders. **Cognitive symptoms:** loss of episodic memory, timing and spatial disorientation, and mood disorders [12,14]
**Parkinson’s disease**	Dorsal IX/X motor nucleus and olfactory bulb [12]	Misfolded α-synuclein, β -sheet amyloid aggregations, Lewy bodies, and Lewy neurites [15]	Noradrenergic neurons, dopaminergic neurons [12,15], especially in the substantia nigra pars compacta [16]	**Nonmotor symptoms:** constipation, visual defects, psychiatric symptoms, cognitive decline, and sleep disturbances. **Motor symptoms:** tremor, rigidity, bradykinesia/akinesia/hypokinesia, and gait disturbances [15]
**Lewy body dementia**	Dorsal IX/X motor nucleus, olfactory bulb, and striatum [16]	Hyperphosphorylated tau, Aβ aggregations, misfolded α-synuclein, βsheet amyloid aggregations, Lewy bodies, and Lewy neurites [15,16,17]	Dopaminergic neurons and cholinergic neurons [13,15,18]	**Nonmotor symptoms:** cognitive impairments, psychiatric symptoms, sleep disturbances, and hallucinations. **Motor symptoms:** bradykinesia, gait disturbances, and hyposmia [16]
**Multiple system atrophy**	Striatonigral and olivopontocerebellar neural and oligodendroglial nucleus [19]	α-synuclein inclusions in Papp–Lantos bodies and glial nuclear, neural cytoplasmic, neural nuclear, and astroglial cytoplasmic inclusions, astrogliosis, and reduced myelination [18]	Oligodendrocytes, dopaminergic neurons, and cerebellar neurons [18,19]	**Autonomic dysfunctions:** erectile dysfunction, lower urinary and cardiovascular symptoms, and dysphonia. **Motor dysfunctions:** tremor, ataxia, postural instability, and oculomotor abnormalities [18]
**Amyotrophic lateral sclerosis**	Brainstem, spinal cord, and skeletal muscles [20]	Aggregations of DNA-binding protein 43 (TDA-43) (in major cases), P-62 aggregations, and misfolded superoxide dismutase [20]	Upper and lower motor neurons [20]	Skeletal and motor cortex atrophy: weakness, cramps, muscle wasting; dysarthria, frontotemporal dementia, and cardiovascular failures [20]
**Multiple sclerosis**	Periventricular area, cortical area, infratentorial area, pons, and spinal cord [21]	Dysregulation of immunological T-cell response that leads to increased inflammation, demyelination, and axonal loss [22]	Oligodendrocytes, motor neurons [22]	Visual failures, spastic paraparesis, ataxia [22], urinary and/or faecal sphincter dysfunction, and tremor [21]
**Huntington’s disease**	Striatum (caudate and putamen), globus pallidus, and nucleus accumbens [23]	CAG triplet accumulations resulting in increasing misfolded huntingtin [23]	Medium spiny neurons of the striatum [24]	Chorea, myoclonus, tics, dystonia, gait, low emotional recognition, and impaired short-term memory, frontal/subcortical impairment [22]
**Creutzfeldt–Jakob disease**	Neocortex, striatum, thalamus, and cerebellar cortex [7]	Prion protein depositions, gliosis, neuropil rarefaction, neuronal loss, and spongiform alterations [7]	Neurons from the neocortex, striatum, thalamus, subiculum, quadrigeminal body, substantia nigra, pontine nucleus, and inferior olivary nucleus [7]	Memory disturbances, myoclonus, visual symptoms, akinetic mutism state, and cognitive impairment [7]

In NDs, protein aggregations spread through vulnerable groups of neurons and drive the development of specific symptoms. On the one hand, the diseases manifest mainly in alterations in higher-order brain functions and cognitive decline resulting from damage to the hippocampus, limbic system, cortex, and neocortex, and on the other hand, in the symptoms of affected thalamus, basal ganglia, cortical areas, and the spinal cord [1]. The latter group of symptoms includes the most detectable alterations; however, by the time symptoms are detected, it is too late to reverse or stop the progression of the disease. NDs present prodromal stages characterised by early and less prominent dysregulations; they can even be asymptomatic. During this period, neuroprotective treatments would be extremely useful. Thus, it is crucial to focus on finding diagnostic methods that use precise biomarkers to detect these pathologies in their earliest stages.

### 1.1. Diagnostic Techniques

All NDs present molecular and structural changes. These molecular changes, called biomarkers, range from protein levels and conformations to inflammation markers and even vesicles. Together with structural and activity changes measured using imaging techniques, these changes can act as cues to aid the diagnostic process [25] (Table 2).

Mendelian inheritance illnesses can be detected by genetic tests that look for a particular mutation, but this is only helpful in isolated pathologies such as Huntington’s disease. Detecting abnormalities in DNA and different types of RNA could help to achieve early diagnoses. However, these abnormalities vary among various NDs and cell types [25]. Genetic diagnosis is more useful in inherited diseases than in the more prevalent idiopathic ones [26].

Noninvasive neuroimaging techniques provide information about the morphological and functional changes that occur in the brain during different stages of NDs. They can be classified into two general groups: nonimaging techniques that measure brain activity (electroencephalography, magnetoencephalography, transcranial magnetic stimulation, etc.) and imaging techniques, such as positron emission tomography (PET), single-photon emission computerised tomography (SPECT), magnetic resonance imaging (MRI), perfusion-weighted imaging, and others [27]. Recent studies have considered diffusion MRI an accurate diagnostic technique because it offers information on the microstructural integrity and complexity of brain tissues in AD and PD [28]. Imaging techniques applied to evaluate the locus coeruleus (LC) in dementias and synucleinopathies have helped to detect these illnesses in the prodromal stages, when this brain area starts to degenerate [29].

**Figure 1:**
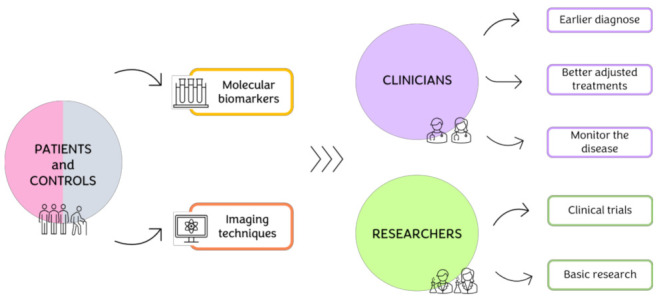
Primary goals for the development and use of molecular biomarkers and imaging techniques in NDs.

Nowadays, one of the main goals in ND research is finding specific biomarkers and developing techniques for their analysis, especially during prodromal phases. Peripheral molecules such as microRNAs, or protein changes in blood cells, are detectable through “-omics” technologies [30]. Others molecules, such as peptides, lipids, and metabolites, could be helpful in differentiating between NDs and are detectable through the analysis of body fluids such as cerebrospinal fluid (CSF) [25]. Despite their diagnostic utility, most molecular biomarkers and imaging tools remain in development and are currently being used predominantly in stage one clinical trials [30]. Advancements in these techniques would allow researchers and clinicians to anticipate, diagnose, and adjust treatment to different disease phases, resulting in more personalised medicine and a better quality of life for patients (Figure 1).

### 1.2. Alzheimer’s Disease and Parkinson’s Disease

In this work, we focus on AD and PD diagnostic methods, since these are the NDs with the highest incidences worldwide. The majority of AD and PD patients show significant symptoms at older ages, confirming that ageing is one of the main risk factors. In patients showing early symptoms, the disease often has a high genetic component. In AD, genetic forms are related to mutations in the *APP*, *PSEN1*, and *PSEN2* genes involved in Aβ peptide processing [31]. In patients with PD, mutations in *LRRK2*, *PRKN*, *GBA*, and *PINK1* predispose them to develop problems in mitophagy and trafficking processes, increasing the impact of the illness at a younger age [32].

According to Braak’s hypothesis, neurodegenerative processes can be classified into different stages that share a common starting point. In AD, tau aggregations appear in brainstem nuclei, involving the LC, transentorhinal region, and the olfactory bulb. At this point, neurofibrillary tangles start to accumulate and spread through the hippocampal formation. Tau pathology reaches the neocortex during the last stages of the disease and finally affects the neocortical association areas [11,14]. In PD, α-syn first begins to aggregate in the olfactory bulb and the dorsal motor IX/X nucleus, leading to the development of Lewy neurites. Lewy pathology spreads through raphe and magnocellular nuclei and the LC. In the middle stage, neurons from the spinal cord and motor areas degenerate, especially in the substantia nigra pars compacta (SNpc). It is at this stage, following impacts on motor function, that the clinical diagnosis is made. In more advanced stages, the transentorhinal region and higher-order sensory association areas in the neocortex become downregulated, reaching premotor and motor areas [12,15].

The most severe stages of AD include difficulties with memory, focusing, orientation, mood, and finally, the inability to complete basic tasks. In the early stages, there is partial memory loss with no clinical manifestations and noncognitive symptoms, such as aphasia, agnosia, apraxia, and visual impairments [33]. In PD, patients develop either motor (rigidity, bradykinesia, tremor, etc.) or nonmotor or prodromal symptoms (depression, constipation, eye alterations, hyposmia, etc.) that can appear more than 10 years prior to diagnosis [15]. The late-stage symptomatic manifestations of these illnesses are the most identifiable using current diagnostic methods. A better understanding of the prodromal or asymptomatic stages of AD and PD would allow detection of these diseases years before the current point of clinical diagnosis and enable intervention with effective therapies to slow down or stop the neurodegenerative process.

## 2. Diagnostic Methods in Parkinson’s Disease and Alzheimer’s Disease

### 2.1. Neuroimaging Methods

As previously noted, new methods for identifying biomarkers that may help clinicians to make earlier ND diagnoses are needed. There is an even greater need for noninvasive detection techniques, such as imaging. In addition to their diagnostic role, these tools can help clinicians to monitor disease stages or even recruit patients for clinical trials.

Access to novel techniques such as MRI, PET, and SPECT, and the discovery of more specific ligands for the dopamine transporter (DAT), has allowed a better understanding of NDs, leading towards the goal of prodromal diagnosis.

#### 2.1.1. Magnetic Resonance Imaging

Brain MRI is useful for both structural and functional imaging. In the case of the former, it allows the exclusion of brain lesions, determination of atrophy hallmarks, and assessment of vascular damage [34]. MRI can reveal patterns caused by neurodegeneration, such as atrophy in the temporal lobe and medial parietal cortex in AD [35]. It also has a key role in the differential diagnosis of PD and atypical parkinsonism, such as parkinsonian-type multiple system atrophy (MSA-P) and progressive supranuclear palsy (PSP).

##### Magnetic Resonance Imaging in Alzheimer’s Disease

Structural Mangnetic Resonance Imaging

In AD, differences in the volumes of some structures can be crucial in diagnosing the disease. Ridha et al. performed an MRI serial study on autosomal dominant mutation carriers and found that patients with familial AD suffered from hippocampal and whole-brain atrophy 5.5 and 3.5 years before diagnosis, respectively [36].

Cortical thickness is also a key biomarker of AD. This feature, particularly in the temporoparietal junction, has been related to more rapid memory deterioration and increased disease progression [37]. Moreover, it may be useful not only for diagnosing asymptomatic patients but also for estimating the severity of their disease [38]. In other studies, the shape of the ventricles has been used to classify patients [39].

Classifying dementia accurately is one of the most challenging aspects of diagnosis, especially in young patients with early manifestation. MRI structural findings could be a valuable tool in this task. For example, Lewy body dementia shows no cortical atrophy, while it can be found in AD; AD shows posterior atrophy before anterior or thalamic, and asymmetric atrophy can be found in frontotemporal dementia patients [40]. Combined with functional MRI findings or molecular biomarker analysis, these features can aid in differential diagnosis.

Functional Magnetic Resonance Imaging

Resting-state fMRI is widely used to detect alterations in the hippocampus, as this region is affected severely by AD. It has been reported that in AD, the hippocampus displays less connectivity. In addition, the precuneus, posterior cingulate cortex, and prefrontal cortex also show altered functional connectivity. Some findings contradict these results, but these indications of higher functional connectivity may be explained by the inclusion of patients with a high level of cognitive functioning or those in the early stages of the disease [41].

The results of task-based MRI studies in AD are less clear. Some studies indicate increased hippocampal functionality [42], while others suggest hyperactivation followed by hypoactivation [43]. While fMRI shows promise in future biomarker detection, it is currently difficult to unify fMRI results between subjects and across cohorts; the results depend on several factors, such as specific fMRI tasks used, the brain regions examined, and the pathological stages of the patient group.

##### Magnetic Resonance Imaging in Parkinson’s Disease

Structural Magnetic Resonance Imaging

A variety of advanced MRI techniques are available for differential diagnosis. An MRI sequence called susceptibility-weighted imaging visualises brain structures—for instance, nigrosome-1 clusters, which are located in the SNpc and appear as characteristic, hyperintense structures under MRI. In PD, the depletion of dopaminergic neurons leads to an increase in the nigrosomes’ iron content, which changes their shape and intensity, making them reliable markers of disease progression. In a recent meta-analysis, structural MRI showed 94% sensitivity and 90% specificity in accurately differentiating healthy adults from idiopathic PD patients [44]. The increase in iron content can also be monitored using MRI in order to distinguish PD patients from healthy subjects [45]. As PD progresses, neuronal death in SNpc leads to an increase in free water. This process is the basis of diffusion-weighted MRI (DWI) techniques, which analyse water motion in tissues that can be used as a diagnostic indicator of PD or atypical PD [46].

Another important biomarker is neuromelanin (NM). This pigment protein is produced as a result of dopamine oxidation and is present in specific brain regions such as the LC and SNpc. PD causes a decrease in NM in these regions. As it shows paramagnetic properties when combined with metals, it represents a feasible candidate for MRI imaging. Recently published studies have focused on using NM MRI to measure the reduction in the area and volume of SNpc. Interestingly, in patients with early-stage disease, there was a greater reduction in the lateral part of SNpc, comparable with the signal attenuation from the LC. Therefore, NM-sensitive MRI sequences could be a remarkable biomarker-based tool for the early detection of PD [29,47].

Furthermore, MRI may be widely used in the differential diagnosis of PD. The literature indicates that MRI hypointensities in the putamen discriminate MSA-P from PD with good sensitivity and specificity rates. In addition, the amount of atrophy in the superior cerebellar peduncle can be used to distinguish PSP from MSA-P and PD [48].

#### 2.1.2. Positron Emission Tomography

Molecular imaging using ionising radioactive ligands such as SPECT and PET allows researchers and clinicians to detect pathological changes in vivo on a cellular or molecular level with high specificity and selectivity. These techniques help to determine brain metabolism and degeneration in patients by detecting transporters, receptors, and enzymes.

##### Positron Emission Tomography in Alzheimer’s Disease

[18F] FDG-Positron Emission Tomography

An analogue of glucose, [18F]-2-Fluoro-2-deoxy-D-glucose (FDG) is a PET tracer that measures the brain’s metabolism. When energy is needed, FDG is phosphorylated and trapped in tissue, mimicking the trajectory of glucose. The trapped FDG rate is measured as a glucose metabolism parameter [34]. It is widely accepted that FDG-PET is useful for differentiating dementias. Although a hypometabolic neocortical pattern can be observed in all types of dementia, care should be taken not to confuse them because of the similarities in their PET profiles. While a medial and lateral temporoparietal pattern with less marked prefrontal hypometabolism is observed in typical AD, a more prefrontal and anterior temporal pattern is seen in frontotemporal dementia. With DLB, there is a dominant parietooccipital hypometabolism with a relatively preserved posterior cingulate. DAT imaging could also be useful in differential diagnosis. Eventually, vascular dementia shows hypometabolism followed by ischemic lesions [49]. Kawasaki et al. warn about the impact of glycemia in FDG imaging and recommend monitoring hyperglycemia and adapting the analysis accordingly when interpreting the images [50].

Amyloid beta-Positron Emission Tomography

In an AD patient’s brain, Aβ accumulates first in the neocortex, then spreads to the allocortex and midbrain regions in phases 2 and 3, eventually appearing in the cerebellum and brain stem in the late stages of the disease [51].

Pittsburgh compound B (PiB), labelled with a C-11 tracer, was the first compound used in amyloid beta-PET imaging. It is derived from thioflavin-T, which binds to Aβ plaques with high affinity. Because of its short half-life, other 18F-labelled ligands were developed [51]. To standardise and unify results across tracers and laboratories, a measuring unit called a “centroid” is used as a means of measuring data collection, biomarker assay, the analysis of data, and the reporting of results [52]. Furthermore, one of the most significant advantages of amyloid beta-PET tracers is that they allow quantification of amyloid deposition in vivo [53].

Tau-Positron Emission Tomography

The advent of selective tau tracers in PET imaging was a significant step forward in the early diagnosis of AD. The most extensively used tracer, Flortaucipir (^18^F), allows researchers to elucidate the relationship between tau and amyloid pathology [39]. Recently, several studies have suggested that tau pathology correlates with atrophy and glucose hypometabolism in affected regions—an association that cannot be found with amyloid plaques [54]. The capability of tau-PET to successfully differentiate between AD and other non-AD neurodegenerative disorders remains controversial. While several studies have highlighted the ability of Flortaucipir (^18^F) to distinguish between types of neurodegenerative disorders with high sensitivity and specificity [55], others have pointed to a low affinity of tau labels to detect non-AD diseases. Marquié et al. explained in 2018 that tau can be present with three or four (3R/4R) microtubule-binding domains and current PET tracers show mixed binding [56]. Even if these tracers are sufficient to detect tau aggregates in typical AD, which present six different isoforms, including 3R and 4R, they show poor binding in non-AD neurodegenerative diseases such as PSP or corticobasal degeneration in which tau aggregates have a preferential accumulation of either 3R or 4R isoforms.

##### Positron Emission Tomography in Parkinson’s Disease

Dopamine Transporter Imaging

Dopamine transporter (DAT) imaging (particularly using 11C- or 18F-PE2I) has been shown to have high sensitivity (87–98%) and specificity (80–100%) in the differentiation of PD from nondegenerative forms of parkinsonism. However, it shows less accuracy in differentiating PD from atypical parkinsonism. It has been argued that striatal DAT loss could be an indication of prodromal PD. In a study by Miyamoto et al., DAT scan predicted the appearance of symptoms of LBD within five years in patients with rapid eye movement (REM) sleep behaviour disorder (RBD) [57].

Other Neurotransmitters

Other strongly correlated transmitters can also be detected. Serotonin can be monitored using the 11C-DASB transporter and is associated with symptoms such as apathy, depression, and anxiety in PD. Noradrenaline can be detected using 11C-MeNER-PET and plays a critical role in sleep disorders and RBD, and cholinergic imaging is also possible with the PET tracer 18F-FEOBV [58].

Brain–Gut Denervation

Current research shows that not only does PD affect peripheral organs, but it may even start at that level. It is well known that in prodromal PD, the gut and heart suffer denervation, which can be detected using molecular imaging techniques. PD patients show reduced uptake of cardiac 123 I-metaiodobenzylguanidine and colonic 11C-donepezil signal and a subsequent loss of FDOPA uptake in the putamen. They may also manifest the FDOPA uptake reduction first, followed by the impairment of the sympathetic nervous system. These findings support the theory that suggests the existence of both body-first and brain-first types of disease [59].

α-Synuclein

The recently developed α-syn radiotracer (18F-ACI12589) showed promising results in identifying MSA-P patients [60]. However, it was not useful for detecting other synucleopathies because of its weak binding to the protein. Developing a tracer for α-syn is especially difficult because of the cytoplasmatic location of the protein, the diverse structure of the fibrils, and the number of aggregates [58].

### 2.2. Molecular Methods

Although neuroimaging techniques have historically been the most used in ND diagnosis, molecular and biochemical markers are currently a main topic in this field of research. Current clinical needs have led to an interest in identifying biomarkers that can be used to discriminate, stratify, and monitor patients in the early stages of the disease. The ideal biomarker should be quantitative, allowing stratification and prediction of the different stages of the disease, as well as measurable in accessible samples such as CSF, blood, or saliva.

#### 2.2.1. Biomarkers in Peripheral Fluids

##### Biomarkers in Alzheimer′s Disease

Accumulation of Aβ is one of the hallmarks of AD, and its levels are already changed years before AD symptom onset, according to the results of PET biomarker scanning [61]. Furthermore, Aβ1-42 and Aβ1-40 have been measured in plasma using mass spectrometry and ELISA assays. Mass spectrometry measurement of Aβ levels in plasma has led to the development of diagnostic tests, one of which has even been approved for clinical use. This test uses Aβ ratio, ApoE proteotype, and patient age to assess the condition of the brain [62]. Other methods can be used to detect amyloid oligomerisation or AD-specific structural changes in plasma peptides relevant to AD [63].

Neurofibrillary tangles containing hyperphosphorylated tau in full-length or truncated forms are another major pathological hallmark of AD, so their study can also provide new and useful biomarkers. Similarly to Aβ, different forms of tau can be measured using various methods in body fluids such as CSF and plasma [64,65]. The first form of tau studied to assess its ability to serve as a biomarker was phosphorylated pTau181, which is increased in plasma according to the severity of pathology. Its levels also correlate with the results obtained using tau-PET and amyloid-PET scanners [66] and with grey matter atrophy [67,68]. This method identifies specific AD neuropathology [67,69,70] and allows discrimination from other non-AD dementias, including other tauopathies that do not present elevated pTau181 levels [67,69,70,71,72]. pTau181 has also been shown to be able to differentiate between patients with mild cognitive impairment who progress to AD and those who do not progress to AD [70,73]. Current data suggest that plasma pTau181 levels offer better diagnostic performance than Aβ42/40, indicating its suitability as a biomarker. Other forms of pTau have been studied as possible biomarkers. For instance, pTau217 can differentiate AD from non-AD dementia with an accuracy of 96%, similar to tau-PET, and performs better than pTau181 [74]. Moreover, plasma levels of pTau217 start to increase 20 years before the onset of the first symptoms, making it a suitable early-stage biomarker candidate, and these changes appear even before those seen using tau-PET [75]. In addition, pTau231 has a discrimination index similar to that of pTau181, but some studies have shown that it appears to change in earlier stages of the disease [76]. Other studies did not confirm its superiority over pTau181 and pTau217 [77].

Unlike Aβ42/40, plasma levels of different forms of pTau increase progressively as AD develops [78], indicating that these forms could be useful not only to achieve earlier diagnosis of AD, but also to monitor the stages of the disease.

Neurofilaments are critical for the growth and stability of axons and for the synaptic function and organisation of the central nervous system (CNS). Two proteins are essential for these structures; one is the neurofilament light chain (NfL) [61]. NfL was the first neurospecific biomarker found [79], and can help to diagnose NDs such as frontotemporal, vascular, and HIV-associated dementia, amyotrophic lateral sclerosis, and atypical parkinsonian disorders [80]. In sporadic AD, NfL is increased in CSF and plasma, which correlates with amyloid-PET and tau-PET as well as with neurodegeneration seen in MRI [81,82]. Although this marker is not highly specific for AD, it has value as an early biomarker of neurodegeneration that, combined with other biomarkers, could be helpful in improving diagnosis.

In addition, glial fibrillary acidic protein (GFAP), specific to astroglial cells, has also been studied with regard to the potential role of reactive astrocytosis in triggering AD pathological changes. GFAP is increased in brain areas where dense Aβ plaques and tau accumulation appear. Plasma and serum GFAP concentrations are higher in patients with Alzheimer´s spectrum symptoms and other pathologies such as frontotemporal dementia and Lewy body dementia [83,84,85]. As is the case for some of the other biomarkers previously mentioned, NfL combined with other techniques or markers could help clinicians to assess early neurodegeneration and diagnose and monitor patients.

Other kinds of molecules can also be used as biomarkers; for example, miRNAs are small noncoding RNAs that regulate gene expression by inhibiting translation or inducing mRNA degradation. The expression of some miRNAs appears altered in the brain, blood, and CSF of patients with AD. These molecules are related to different functions such as neuroinflammation (miR-125b and miR-146a), cell cycle regulation (miR-26b, miR-107, and miR-125b), and neuronal cell cycle and apoptosis (miR-34a) [86].

##### Biomarkers in Parkinson´s Disease

The appearance of misfolded α-syn in the brain has long been studied as a pathological feature of PD. Therefore, it has been the focus of research to identify and validate PD biomarkers. The presence of α-syn can be found in fluids such as CSF, blood, and saliva, as well as in exophages, colon, and peripheral tissues such as the skin, among others [87]. The presence of α-syn in these samples suggests α-syn as a good candidate for use as a PD biomarker, so its levels have been measured using different techniques and samples.

Total values of α-syn in CSF have been studied as a possible biomarker. However, the heterogeneity of results and techniques, as well as different patient characteristics, currently make it impossible to establish a correlation between CSF total α-syn and the PD diagnosis [88,89]. Nevertheless, α-syn can be useful as a marker for synucleinopathy [64] and in assessment of PD patients, as higher levels of total α-syn in CSF correlate with a faster progression of the condition [90]. Techniques such as protein misfolding cyclic amplification (PMCA) and real-time quaking-induced conversion (RT-QuIC) have allowed researchers to determine levels of α-syn pathogenic aggregates in biofluids. Specifically, RT-QuIC proved to be a good method to distinguish confirmed PD patients from controls with 95% sensitivity, and Lewy body dementia from controls with 92% sensitivity. Both methods showed 100% specificity in CSF samples [91]. Correlation between levels of these pathogenic aggregates of α-syn and the Hoehn and Yahr scale was established in several studies [92], making it possible to monitor PD patients.

Phosphorylated α-syn (pS129) is associated with the aggregated forms of α-syn and makes up 90% of the α-syn present in LBs. Thus, it is a good candidate for biomarker research. To date, few studies have measured pS129 α-syn in CSF, showing that pS129 represents approximately 12–15% of the total α-syn in CSF [93]. However, correlations between pS129 and pathology are still unknown and more research would be necessary to assess the accuracy of this marker [88].

These CSF findings raised questions about whether it might be helpful to test α-syn in other fluids with easier access. With an easy and minimally invasive extraction procedure, blood is one of the best candidate samples for biomarker searches. Total and oligomeric forms of α-syn have been measured in plasma and serum; pS129 was found to be increased in plasma in PD patients [94,95]. As with CSF, however, the quantification of total α-syn in plasma and serum has produced significant heterogeneity of results because of the different techniques used in the assays [87].

Levels in plasma and serum are difficult to assess because of the contamination of samples with red blood cells, which contain a great proportion of the total α-syn [96]. For this reason, other fluids, such as saliva, could potentially be used, as saliva samples are obtained easily and are not contaminated with blood cells. Additionally, LBs have been found in salivary glands [97,98]. Several studies showed lower total salivary α-syn in PD patients than in controls [99,100,101,102] while in other studies, total α-syn in saliva could not differentiate between PD and controls [103]. On the other hand, oligo α-syn was higher in PD patients, and significant correlations with the Hoehn and Yahr scale of pathology were also found [102,104]. Thus, the presence of α-syn in peripheral fluids is a good PD biomarker candidate and could also be used to classify patients; however, a consensus regarding quantification methods is necessary to obtain more reliable results.

As is the case for AD, miRNAs are promising biomarkers for PD. In particular, dysregulation of miR-30, miR-29, let-7, miR-485, and miR-26 has been observed in the brain, CSF, and blood cells of PD patients. Standardised protocols for sample collection and processing must be established prior to standardising the use of miRNAs as biomarkers in NDs [105].

In conclusion, there are several candidates for neurodegeneration biomarkers, and some could discriminate efficiently between different NDs, allowing early diagnosis and patient monitoring.

#### 2.2.2. Inflammation in Neurodegenerative Diseases

Inflammation is a common feature of many pathologies, including NDs. Neural damage coexists with neuroinflammation, which makes it possible to find alterations in molecule inflammation levels and immune cell populations in blood and CSF. These changes can contribute to the progression of disease pathology, and some are related to genetic modifications that have already been associated with some NDs.

Researchers have studied the implications of neuroinflammation in PD. Using PET ligands, it is possible to measure and trace microglial activation in PD patients, which appears to be augmented in PD-related brain areas. However, this does not correlate with the stages of the disease [106], indicating the need for other markers and the study of immune dysregulation in the periphery.

PD causes immune dysregulation in the periphery and brain; this leads to the upregulation of inflammatory cytokines. In the brain, levels of pro-inflammatory cytokines are increased; in some cases, their levels in the brain correlate with blood levels, as happens for TNF, IFNγ, IL-1β, IL-6, IL-2, and CCL2 [107,108]. The cytokines that have received the most attention are IFNγ and TNF. TNF is increased in serum, CSF, and brain [109,110,111,112], consistent with its role in nigral degeneration. In addition, TNF signalling neutralisation attenuates dopaminergic neuron death in rodent models [109,110,111]. Furthermore, PD blood monocyte populations show increased proliferative capacity compared with controls [112]. An increase in IL-17, IL-4, and IFNγ production by T cells has also been reported, and the target of these cells seems to be α-syn [113,114,115].

Neuroinflammation is common in AD, causing astrogliosis, microglial activation, and an increase in the release of inflammatory cytokines, such as IL-1β, IL-6, TNFα, and TGF-β. Furthermore, peripheral immune cells such as macrophages or T cells are present in AD [116]. In the CNS, reactive astrocytes are found in postmortem analysis of AD patients’ brains, specifically in areas with amyloid plaques [112,117]; when astrocytes are activated, they produce inflammatory cytokines as well as reactive oxygen species. The production of pro-inflammatory cytokines could also provide potential biomarkers since, in many cases, blood levels correlate with the levels in CNS.

#### 2.2.3. Exosomes

Exosomes are small membrane microvesicles (MVs) derived from endosomes. They have a diameter ranging from 30 to 150 nm and are composed mainly of lipids and proteins enriched with lipid rafts [118]. This type of MV is released from most cell types into the extracellular space and is considered responsible for removing debris [118,119].

Exosomes have been found in numerous body fluids, such as blood, saliva, amniotic fluid, breast milk, urine, CSF, sperm, synovial fluid, lymph fluid, and follicular fluid. They are carried into cells by travelling through body fluids and participate in different physiological and pathological processes. Their function varies, mainly depending on the origin of the cell or tissue from which they are released [120]. The physiological function of these MVs is to transport biomolecules between different cells, acting both locally with neighbouring cells and via the bloodstream, moving through organs and tissues; they are thus considered a mechanism of paracrine, autocrine, and endocrine communication [121,122]. In physiological conditions, exosomes contribute a vast number of functions in tissue repair [123,124,125], inflammatory processes, homeostasis [119,126], angiogenesis [127,128,129], synaptic plasticity, and neuroprotection and neuronal cell survival [130,131].

##### Exosomes in Neurodegenerative Diseases

Because of the role of exosomes in the regulation of molecular pathways in malignant neoplasms [132], exosomes have been extensively studied in the context of tumour development [133]. Exosomes have received more attention in recent years because of their secretion in various cells of the CNS and their role in transporting misfolded or aggregated proteins, which are a key element in the progression of neurodegenerative diseases [134,135,136]. In AD, it has been proposed that exosomes transfer pathogens such as APP, which leads to Aβ deposition in the brain. Other exosomal proteins, such as tau [134], Alix, and flotillin 1, have been found to accumulate in the AD brain [136]. Still, several findings suggest that these exosomes have a neuroprotective role, removing toxic oligomeric species in the exome lumen [133,136] or capturing Aβ, thus reducing Aβ load in the brain [134].

In PD, exosomes have been shown to transfer α-syn protein into normal neuronal cells, leading to the formation of aggregates and induction of receptor cell death [137,138].

Transmission of misfolded α-synuclein to neurons and astrocytes by neuronal exosomes, in addition to the transport of toxic α-syn oligomers into the extracellular environment, induces inflammation and cell death [139]. Additionally, exosomes rich in major histocompatibility complex class II (MHC-II) and pro-inflammatory cytokines such as TNF-α, which induce neuronal apoptosis [140], and mitochondrial DNA can trigger inflammatory reactions, thus participating in disease propagation [139].

It has been shown that secreted exosomes can have toxic and neuroprotective effects on the nervous system [141]. These MVs can remove misfolded proteins that hinder neural stem cell formation [142]. On the one hand, increased release of α-syn-containing exosomes reduces intracellular levels of a-syn protein and may explain the survival of substantia nigra neurons in sporadic PD patients overexpressing PARK9/ATP13A2 [136]. Brain neurons and glial cells can also eliminate and reduce harmful metabolites and proteins in cells via extravasating exosomes [139]. On the other hand, α-syn oligomers associated with exosomes have been found to increase the likelihood of cellular uptake, with greater subsequent neurotoxicity than free α-syn oligomers [138].

##### Exosomes as Biomarkers

Exosomes are characterised by their size, morphology, flotation density, and the presence of marker proteins such as Alix, TSG101, flotillin 1, HSP70, and CD9. The specific profiles of these MVs can reflect their cellular origin and the physiological state of the secreting cell, reflecting cellular processes which can be used as biomarkers for various diseases [143]. As discussed above, these exosomes are found in body fluids, making them ideal noninvasive biomarkers for disease diagnosis and prognosis [135]. Furthermore, exosomes are well tolerated by the human body and are capable not only of penetrating cell membranes but also of potentially targeting specific cell types [144]. Exosome isolation techniques include ultracentrifugation, ultrafiltration, chromatography, polymer-based precipitation, and antibody-coupled magnetic affinity beads, with ultracentrifugation being the most widely used technique because of its high processing capacity [145,146].

Regarding fluids used for evaluation of biomarkers in ND, we mainly found reports of CSF and blood. It has been shown that Aβ42, T-tau, and P-T181 exosomes derived from neurons in peripheral blood can reflect pathological changes in AD in the brain [147], and decreased exosomal miRNA expression has been observed in the CSF of AD patients. Similarly, decreased miRNA levels in PD have also been found in patients’ CSF [148], and it has been reported that it is possible to distinguish between patients with PD and multiple system atrophy by measuring α-syn in blood exosomes [149]. Finally, in other NDs, such as HD and ALS, significant differences in exosomal miRNA expression in serum and CSF have also been detected [148].

In addition to the use of these fluids for investigation of exosome biomarkers, salivary exosomes have become a topic of prime importance because of the advantage of noninvasive sampling. Studies on salivary exosomes in PD have reported that the content of α-synOlig and α-synOlig/α-synTotal is higher in saliva-derived extracellular vesicles in PD patients than in healthy controls [150] and that a higher concentration of neuronal salivary exosomes has been found in PD patients compared with healthy controls. In addition, the levels of L1CAM and α-syn proteins were also elevated in PD patients [151].

Exosomal components reveal the secreting cell’s biological state and can provide information about the state of health of an organ or tissue. Lipids are one group of these components, and the brain is one of the organs with the highest concentrations of lipids. It has been shown that the CSF of patients with multiple sclerosis (MS) is enriched with acid sphingomyelinase which transforms sphingomyelin into ceramides, inducing axonal damage and mitochondrial dysfunction in this disease and suggesting that lipid alterations of exosomes may be due to pathological conditions in NDs [152]. Interest in using exosomal lipids as biomarkers for this purpose has been growing in recent years, and future comprehensive studies will be indispensable [152].

Exosomes have a high capacity to target tissues or cells and penetrate biological barriers (such as the blood–brain barrier), and thus offer advantages for natural drug delivery. Although methods for introducing RNA and proteins into exosomes are still developing, the ability to carry both proteins and genetic material is another advantage that makes exosomes an attractive drug delivery system [144]. However, natural exosomes can have problems, such as the possibility of being rapidly eliminated by the body, which reduces the effect of the treatment. Therefore, they are often modified to form artificial exosomes capable of transporting their cargoes across the blood–brain barrier and conferring an active biological effect exactly on the target cells [143].

#### 2.2.4. Autoantibodies

Autoantibodies or natural antibodies consist of immunoglobulins that react with self-antigens both in healthy individuals and in patients with autoimmune diseases, since the immune system sometimes fails to distinguish between self-antigens and non-self-antigens [153,154]. Autoantibodies are mainly produced by a small subset of B lymphocytes and can consist of proteins, nucleic acids, carbohydrates, lipids, or various combinations of these biological materials [155].

Each specific autoantibody can simultaneously have several isotypes and subclasses present that potentially influence the pathophysiology of a disease. Human B lymphocytes express five types of immunoglobulin: IgM, IgD, IgG, IgA, and IgE. Each isotype and subclass exerts a different function, allowing adjustment and shaping of the immune response and the elimination of a wide variety of pathogens [156].

Autoantibodies induce disease via a multitude of pathophysiological pathways, and there may be different mechanisms contributing to clinical manifestation within a single disease [157]. Self-reactive antibodies are not necessarily pathogenic, as they can be found in healthy populations, although they cannot be seen in high concentrations and, for the most part, do not cause damage or attack the host [157]. These types of autoantibodies can mediate both systemic inflammation and tissue injury as well as protect against autoimmune diseases [153,158].

##### Autoantibodies in Neurodegenerative Diseases

In general, the production of autoantibodies is a feature of most autoimmune diseases, including rheumatoid arthritis or type 1 diabetes [158]. Although the underlying mechanisms that explain the production of autoreactive B cells and autoantibodies in patients with autoimmune diseases remain unclear, it has been shown that autoantibodies can cause the deposition of immunocomplexes in various organs, thereby activating the complement system or activating immune cells, leading to severe inflammatory damage. In addition, they can also cause direct target tissue damage through antibody-dependent cell-mediated cytotoxicity [158,159].

However, the presence of autoantibodies is not exclusive to autoimmune disease. The clinical profile of antibody-mediated cognitive impairment has led to a particular interest in the potential pathogenicity of neuronal autoantibodies in neurodegenerative dementias [160], and several studies have correlated autoimmune diseases with neurodegenerative diseases such as AD [161] or PD [162], so the interest in and knowledge of CNS autoantibodies are increasing [160].

Several studies have shown that antibodies have both a pathologic and protective effect in AD. An interaction between IgG and tau protein has been demonstrated, supporting a pathological role of Ig in the disease, and supporting the theory that blood–brain-barrier dysfunction in AD allows autoantibodies to access targets in the brain, leading to autoimmune-induced neuronal cell death. On the other hand, it has been shown that IgGs are detected mainly in microglia and some neurons but not in astrocytes, showing that Igs could prevent Aβ pathology by increasing phagocytosis by microglial cells, resulting in increased Aβ clearance [163].

In PD, autoantibodies also play a bifunctional role in the pathogenesis of the disease. Studies have revealed that some pigmented dopaminergic neurons in the SNpc of PD patients have more IgG than those of healthy controls, in addition to IgG colocalising with α-syn. Notably, antibodies against α-syn protect against PD by neutralising aggregate accumulation and, thus, synaptic loss [163]. Likewise, some studies have reported a marked loss of dopaminergic neurons in the substantia nigra of rats treated with plasma antibodies isolated from PD patients, while those treated with antibodies from healthy controls showed much less neuronal damage [164].

Thus, in neurodegenerative diseases, humoral autoimmunity may not be exclusively present or absent, but rather may subtly alter the progression of protein aggregation, misfolding, and degeneration [165].

##### Autoantibodies as Biomarkers

Knowing the effects of autoantibodies on neuronal function and neurodegenerative diseases, their prevalence in the patient population, and their titers in patients could provide useful biomarkers with which to measure the risk of developing a neurological disease [165].

The following correlations are being studied: LGI1 antibodies and [166] voltage-gated potassium channels and frontotemporal dementia [167] or unspecified reversible dementia [168], GFAP-specific antibodies and PD [169], GABAB receptor-specific antibodies and amyotrophic lateral sclerosis (ALS) [170], cell-surface-binding antibodies and Creutzfeldt–Jakob-like disease [171], and IgLON5-specific autoantibodies and a sleep disorder with abnormal movements and cognitive decline [165,172].

Many of these autoimmune diseases are diagnosed on the basis of serological detection of autoantibodies present in the patients. The best-known antibodies among those detected are antinuclear antibodies, which are present mainly in autoimmune diseases such as systemic lupus erythematosus and systemic sclerosis [173]. When autoantibodies cannot be detected via the usual methods, novel techniques are used, such as determining specific autoantibody isotypes in suspected cases of rheumatoid arthritis [174] or using a keratinocyte-binding assay in suspected cases of pemphigus [175]. In addition, autoantibodies are also found in certain diseases, such as NDs [176] and chronic obstructive pulmonary disease [177], which are now being recognized as mediated by autoantibodies.

## 3. Future Challenges in Personalised Diagnostics

Despite the considerable advances that have been achieved in recent years in diagnostic methods for NDs, these methods still suffer from significant limitations that restrict their diagnostic efficacy, especially in the early stages of the disease. As mentioned above, molecular biomarkers have a set of strengths that offer great potential for the early diagnosis of NDs, such as their high sensitivity in the early stages of disease, their easy measurement in fluid samples, and their simple implementation in screening systems for the identification of high-risk patients, among others. However, their ability to cross the blood–brain barrier and the slight changes that arise under pathological conditions limit their diagnostic capability, leading to false positive or false negative results. Moreover, several of these molecular biomarkers are involved in acute-phase responses and thus are present in the early stages of multiple different diseases, reducing their predictive power. These constraints make it necessary to confirm diagnoses with other techniques, such as neuroimaging. Noninvasive neuroimaging technologies are widely used to identify regional and global brain atrophy and can differentiate between different types of dementia in the advanced stages of the disease. However, they show reduced sensitivity and specificity in the prodromal phases, and their late application restricts treatment possibilities in NDs (Table 3).

In response to these constraints, substantial efforts are being made towards both the discovery of new biomarkers and the optimisation of measurement technologies. In this sense, combining high-throughput and high-sensitivity technologies with artificial intelligence tools for multivariate analysis of multiple biomarkers opens a promising window of opportunity not only for early-stage detection but also for stratification and monitoring of patients with NDs. However, significant challenges remain to be solved, such as the difficulty of standardising and validating biomarkers across different laboratories and studies, the ethical considerations surrounding biomarker tests and their use in decision-making processes, and the dependence on technological advances and the availability of equipment. In conclusion, important challenges are still ahead in diagnosing NDs, but combining sensitive technologies with more specific biomarkers could lead to a remarkable improvement in this field. Moreover, developing predictive models based on neuroimaging data and peripheral biomarkers offers good noninvasive options to complement current methods based on clinical evidence. The advancements achieved in this area will contribute not only to more accurate diagnoses, but also to the stratification, monitoring, and prognostic evaluation of patients, leading to more efficient treatment and improvement in patient quality of life.

## Figures and Tables

**Table 2:** Summary of the main molecular biomarkers and imaging techniques used in the diagnosis of Parkinson’s disease and Alzheimer’s disease.

DIAGNOSTIC TOOLS IN NEURODEGENERATIVE DISORDERS				
MOLECULAR BIOMARKERS		IMAGING TECHNIQUES		
Biomarkers in peripheric fluids	Alpha-synuclein in Parkinson’s Disease (PD)	Magnetic Resonance Imaging (MRI)	Structural MRI	
	Amyloid-β and tau in Alzheimer´s Disease (AD)			
			Functional MRI	
Inflammation markers				
		Positron Emission Tomography (PET)	PD	DAT imaging
Exosomes				Neurotransmitters
				Brain-gut denervation
				Alpha-synuclein
Autoantibodies			AD	[18F] FDG–PET
				Amyloid-beta
				Tau

**Table 3:** Strengths, weaknesses, opportunities, and challenges of diagnostic methods for neurodegenerative diseases.

Molecular Biomarkers	Neuroimaging Techniques
**Strengths**	
Can be easily and minimally invasively collected through blood, urine, or saliva samples; Can be measured in the early stages of the disease, potentially enabling early diagnosis and treatment; Can be tested in large populations for screening and identification of at-risk individuals; Can provide information about specific pathological processes and pathways involved in the disease; Can potentially be used to monitor disease progression and response to treatment.	Noninvasive techniques; Ability to monitor brain regions and identify and quantify diagnostic and candidate biomarkers of dementia progression; Widely used and validated method; Capable of measuring both regional and global brain atrophy; Can differentiate between different types of dementia; Can be combined with other techniques to improve accuracy.
**Weaknesses**	
Dilution of brain-derived proteins and metabolites in blood samples; Limited sensitivity and specificity in some cases, leading to false positive or negative results; May not accurately reflect pathology in the brain due to the blood–brain barrier; May not be specific to a particular ND, leading to difficulty in differentiating between diseases; Limited understanding of the underlying mechanisms and significance of some biomarkers.	Expensive and not widely available in many hospitals and clinical centres; Limited in detecting biomarkers closely related to brain pathogenesis because of the blood–brain barrier; Limited sensitivity and specificity in some cases, especially during prodromal phases; Late application, low treatment possibilities; Overlap between NDs.
**Opportunities**	
Continued development and optimisation of biomarker measurement technologies and assays; Integration of multiple biomarkers into panels for improved diagnostic accuracy; Exploration of new biomarkers, such as microRNAs and post-translational modifications; Potential use in personalised medicine and targeted treatment approaches.	Ability to predict early conversion to dementia in patients with mild cognitive impairment; Potential for early diagnosis and treatment of Alzheimer’s disease; Potential for monitoring disease progression.
**Challenges**	
Limited financial resources for biomarker research and development; Difficulty in standardisation and validation of biomarkers across different laboratories and studies; Ethical considerations surrounding biomarker testing and use in decision-making processes.	Limited accuracy in some cases; Competition with other diagnostic techniques; Dependence on technological advances and availability of equipment.

## Data Availability

Not applicable.
